# Diuretic Resistance Associated With Heart Failure

**DOI:** 10.7759/cureus.21369

**Published:** 2022-01-18

**Authors:** Elham Shams, Sabrina Bonnice, Harvey N Mayrovitz

**Affiliations:** 1 Osteopathic Medicine, Nova Southeastern University Dr. Kiran C. Patel College of Osteopathic Medicine, Davie, USA; 2 Medical Education, Nova Southeastern University Dr. Kiran C. Patel College of Allopathic Medicine, Davie, USA

**Keywords:** glomerular filtration rate, angiotensin-converting enzyme, sodium retention, over-prescription, diuretic resistance, renin-angiotensin-aldosterone system, pathophysiology, renal failure, heart failure

## Abstract

Fluid retention is common in patients with heart failure (HF) and diuretics are a major source of management and stabilization. Diuretic resistance (DR) is defined as a failure to achieve therapeutically desired congestion relief despite using an appropriate diuretic dose. In this study, the underlying mechanisms of DR in HF patients are described and several currently available evidence-based strategies to mitigate this condition are provided. Specific aims were to investigate how DR occurs in patients with HF, how HF medications interfere with diuretic treatment, and what alternative methods are available for patients with DR while undergoing treatment for HF. Results provide several rationales for novel strategies used to help reach a state of euvolemia where there is a proper amount of blood in the circulatory system. These include reducing sodium intake, changing the timing of drug administration, and modifying diuretic dose.

## Introduction and background

Heart failure (HF) is responsible for about one million hospitalizations annually in the United States, which is a number that is rising [1]. Fluid retention is common in patients with HF and diuretics are a major source of management and stabilization [2]. Measuring the amount of sodium and water, frequent weight monitoring, and refraining from medications such as non-steroidal anti-inflammatory drugs (NSAIDs) are crucial in preventing salt and water retention [3]. In HF patients, the prevalence of diuretic resistance (DR) is estimated as 20%-30% [4]. In this review, the key mechanisms of DR in HF patients are described and numerous evidence-based approaches to mitigate this condition are provided. The specific aim was to investigate the following questions: (1) How does DR occur in patients with HF? (2) Can HF medications interfere with diuretic treatment? (3) What alternative methods are available for patients with DR while undergoing treatment for heart failure?

## Review

To consider the previous questions in context, it is first useful to examine the main physiological components of loop diuretic actions, as these are the most commonly used diuretics in the treatment of HF [3]. Loop diuretics reversibly inhibit symporters at the thick ascending limb of the loop of Henle where one-third of filtered sodium is reabsorbed. Loop diuretics are often preferred over other diuretics for HF therapy since most sodium reabsorption occurs at the thick ascending limb. Thus, loop diuretics can have a greater effect on volume regulation. Other diuretics such as thiazides and potassium-sparing diuretics act on the distal ascending limb and the collecting ducts, respectively, where less sodium reabsorption occurs [3].

Physiology of loop diuretics

Loop diuretics affect ion transport directly by binding to the translocation pocket at the extracellular surface of sodium-potassium-chloride cotransporters (NKCCs) [5]. These diuretics act at the apical surface of the thick ascending limb cells along the loop of Henle (NKCC2, gene SLC12A1), thereby inhibiting the sodium-potassium-chloride symporter. Loop diuretics, such as furosemide, bumetamide, and torasemide, also inhibit this same symporter at the apical membrane of macula densa cells where they inhibit tubuloglomerular feedback and also stimulate renin secretion [6]. The diuretic effect may help relieve congestion in HF patients. Contrastingly, due to the loop diuretic-induced activation of the renin-angiotensin-aldosterone system (RAAS), there may also be elevated plasma renin activity that causes angiotensin II to increase. This can further inhibit tubuloglomerular feedback, thus decreasing the glomerular filtration rate (GFR) [6].

Loop diuretics have intricate effects on renal and systemic hemodynamics, which are influenced by the dose and route of administration and chronicity of use. As mentioned, loop diuretics activate the RAAS which by itself would tend to increase systemic blood pressure and sodium and water reabsorption. Loop diuretics also increase vasodilatory prostaglandins that cause pressure within the proximal tubule to increase [7]. The consequences of these sometimes counteracting effects are that high-dose intravenous (IV) loop diuretics can decrease or increase arterial pressure, and decrease renal blood flow [8]. Higher doses of the diuretic may either override the resistance and produce a diuretic effect, or it may continue to fail. It is challenging to predict, in an individual patient, which effects will prevail.

Causes and mechanism of diuretic resistance in HF

Decreased renal function and delayed peak concentration of loop diuretics can be identified as causes of DR. For instance, one quarter of cases with renal failure are found to have HF [9]. Thus, it is common to see patients with HF and impaired renal function. Delayed peak concentrations can be attributed to the increased number of competitive anions binding to organic anion transporter (OAT) receptors, which inhibit the diuretic from binding to its site of action. This prevents the diuretic’s efficacy by prolonging its peak concentration [6].

Another cause of DR is structural changes in cells, also known as the “braking phenomenon”. Based on the previous literature, studies in rats demonstrated both hypertrophy and hyperplasia in epithelial cells of the distal convoluted tubule after chronic use of loop diuretics [10]. Additionally, prolonged exposure to loop diuretics leads to nephron remodeling with hypertrophy of the distal tubular cells, which, in turn, may alter the diuretic response due to a compensatory increased sodium reabsorption [11]. This results in the braking phenomenon in which chronic treatment with loop diuretics causes decreased blood sodium levels [10]. Thus, chronic use of diuretics increases resistance towards these diuretics.

Effects of HF medication in DR

Another cause of DR is the use of NSAIDs [12]. NSAIDs can decrease renal blood flow and thereby decrease the GFR due to vasoconstriction of renal afferent and efferent arterioles. The decreased GFR reduces tubular secretion of loop diuretics, causing diuretic ineffectiveness. NSAIDs can exacerbate the situation by interfering with prostaglandin E2 (PGE2) synthesis thereby reducing PGE2’s stimulation of sodium and water excretion. NSAIDs decrease the vasodilation of renal vessels by inhibiting prostaglandin synthesis [13]. NSAIDs block the cyclooxygenase enzymes (COX-1 and COX-2) that produce PGE2, inhibiting its production. Thus, the use of NSAIDs can exacerbate DR by both decreasing renal blood flow and inhibiting prostaglandin synthesis.

Chronic low-dose aspirin may also disrupt platelet PGE2 production, decreasing vasodilation and diminishing diuretic effects by irreversibly inhibiting COX-1 and COX-2 [14]. In addition, there are indications that even low doses of aspirin might neutralize diuretic effects of angiotensin-converting enzyme (ACE) inhibitors by impeding PGE2 production and promoting increased renal vascular resistance [15]. It has been argued that in patients with HF who have renal insufficiency, aspirin and other antithrombotic agents should be carefully administered to mitigate possible disruption of PGE2 synthesis [16].

Pharmacokinetics and oral bioavailability in DR

Different pharmacological agents are shown to have significant differences in oral bioavailability. In the family of loop diuretics, the oral bioavailability of torasemide and bumetanide typically exceeds 80%, whereas that of furosemide is substantially lower, at approximately 50% [17]. Evidence shows that the consumption of loop diuretics with food decreases their maximal plasma concentration by almost 50%, nevertheless extending the time of their presence in the blood [17]. Because loop diuretic plasma concentrations must surpass a certain threshold to stimulate natriuresis, food consumption with medication exacerbates the possibility of DR. In addition, in patients with HF who have marked volume overload, the absorption of loop diuretics may be compromised as a result of poor intestinal perfusion and various bowel diseases [18].

Due to the significant volume overload in hospitalized HF patients, the administration of IV loop diuretics is favored over oral intake due to oral bioavailability and gastrointestinal reabsorption [19]. There is no significant difference between bolus versus continuous administration regarding effective diuresis; however, the outcome is linked to a more effective neurohumoral activation. The advantage of continuous administration of loop diuretics is that there is no evidence of post-diuretic sodium retention, yet this can be controlled by providing bolus loop diuretic at six- to eight-hour intervals if the patient continues to have symptoms of congestion [20].

Co-morbidities as a contributing factor to DR

DR is more common in patients with a higher use of diuretics, such as patients with HF. However, other co-morbidities may also play a role in the development of resistance towards these drugs. Chronic kidney disease (CKD) and HF share a great number of physiological pathways along with numerous risk factors [21]. Furthermore, patients suffering from right ventricular dysfunctions and congestive states due to pulmonary disorders may also develop DR due to renal perfusion and oxygenation issues [22]. Other diseases, such as diabetes mellitus (DM), contribute to hypoperfusion and organ damage as exemplified by diabetic nephropathy [23]. Patients with diabetic nephropathy may develop nephrotic syndrome [24]. Nephrotic syndrome is characterized by a resistance towards diuretics, and it is also present in HF patients who have developed DR [25]. Thus, in patients with both HF and DM, these conditions can simultaneously lead to nephrotic syndrome and DR [26]. In a study evaluating DR and its relationship to other comorbidities such as hypercholesteremia, DM, valvular disease, CKD, and cancer, patients suffering from any of these diseases along with DR had a worse prognosis than those without DR [27].

Prediction of diuretic resistance in HF patients

Predicting DR before it becomes clinically impactful may help prevent a worsening prognosis in patients with HF [28]. A natriuretic response prediction equation (NRPE) may be used to monitor natriuretic response in HF patients [29]. It is used to measure urinary sodium output (NAout), in mmol, based on data obtained from a urine sample taken two hours after loop diuretic administration [30]. Since creatinine has limited reabsorption and secretion in the tubule, the serum creatinine to urine creatinine (CrS/CrU) ratio reveals the sodium concentration level in the tubules. Therefore, the rapid rate of urine formation (ml/min) can be derived from the serum to urine creatinine ratio and the estimated GFR (eGFR) product [30]. Multiplying this result by the urine sodium concentration (NaU) provides conversion from the instantaneous rate of urine formation to sodium excretion (mmol/min). The equation is, NAout = eGFR x (BSA/1.73) x (CrS/CrU) x 60 min x 2.5 hours x (NaU/1000 ml), where BSA is the body surface area. The various constants in this equation can be understood with the following. The majority of natriuresis happens quickly after the administration of a bolus IV loop diuretic, and natriuresis is accomplished in six hours. The instantaneous rate of sodium excretion is converted into a cumulative sodium output by multiplying by 2.5 hours [31]. The time of 2.5 hours in the equation was chosen to convert peak instantaneous natriuresis into cumulative natriuresis since most natriuresis occurs soon after IV diuretic administration. The 1.73 m^2^ constant normalizes BSA to an assumed normal value. If patients who are responding sub-optimally to diuretics just an hour or two after a loop diuretic is administered are detected, repeat dosing could occur much more quickly, which offers benefits concerning outcomes ranging from faster symptom relief to perhaps a reduction in the length of hospital stays or improvement in overall decongestion [30].

Prognosis of diuretic resistance in HF

In patients with HF, estimating mortality risk and assessing disease severity are essential for clinical management and special interventions [4]. Diuretic resistance in patients is shown to contribute to worsening HF during an inpatient stay, prolonged lengths of stay, and likely increased mortality along with consumption of more resources in comparison to those who respond adequately to the initial diuretic administration [32]. Since diuretic resistance can be overcome by increasing doses or drug combination therapies, the assessment of congestive heart failure (CHF) severity may be insufficient without considering therapy intensity [33]. In a retrospective study of 1153 patients, the relationship of CHF treatment and other baseline variables to the mode of death and mortality was evaluated. It was found that increased doses of loop diuretics including furosemide >80 mg or bumetanide >2 mg daily were independent predictors of total mortality and/or cause-specific mortality in patients with advanced CHF [4]. The results suggest that diuretic resistance, clinically defined as a high-dose requirement, should be considered to determine the prognosis in chronic CHF patients.

Overcoming diuretic resistance with alternative methods

Several approaches are available to help deal with diuretic resistance.

Increased Dose

One approach to dealing with DR is to increase the dose allowing needed amounts of the diuretic to reach the urinary site of action in patients with HF [34]. One study showed the efficacy and potency of high-dose furosemide (250-4000 mg/day, given orally or intravenously) in 35 patients with severe CHF and reduced renal function. No significant side effects were reported but all patients had a reduction in weight and had relief of symptoms [35]. However, furosemide efficacy in patients with renal impairment is impeded by reduced activity of the OATs and decreased renal blood flow that may lead to diminished concentrations in the renal tubule [34]. Therefore, in most cases, increased dosage will overcome DR in patients with HF, while it will not resolve DR in patients who have reached renal impairment.

Use of Thiazide-Like Drugs in Combination With Loop Diuretics

Thiazide-like diuretics have been used with loop diuretics as a combination therapy option for patients diagnosed with DR in HF. All combinations of any thiazide-like diuretic with a loop diuretic have proven to work just as efficiently in reducing fluid retention in patients with DR in HF [36]. A recent small cohort study compared oral metolazone and IV chlorothiazide, the two most common thiazide-like diuretics, as add-ons to loop diuretic therapy in patients with DR. Each helped improve patient status and there were no statistical differences between their efficacy or safety [37].

Drug Infusion Route

One way to reduce the chance of DR in HF patients is by changing the drug administration route from oral to IV using a bolus infusion [37]. Shortly after administering an IV bolus, patients with congestive symptoms noticed improvement due to reduced pulmonary artery pressure and increased venous capacitance postulated to be due to effects of increased prostaglandin release [38]. This method of management was found to be a successful form of therapy in HF patients who are refractory to high-dose oral diuretic therapy [34]. Delivery of the medication via this route bypasses the gastrointestinal tract and overcomes problems related to a delay in absorption [34]. A side effect of this form of administration in high doses is the possibility of ototoxicity development, specifically in patients concurrently receiving other ototoxic drugs, such as aminoglycosides (e.g., gentamicin) [39]. It is reported that the ototoxic effect can be mitigated by slowly administrating furosemide instead of a bolus when doses are greater than 80 mg to avoid a sudden increase in the peak serum concentration [40].

Potential Use of Urea to Overcome Antidiuretic Resistance

Although the use of urea in HF is very limited in clinical practice, the oral administration of urea has been proposed as an additional treatment to correct hyponatremia [41]. HF triggers increased antidiuretic hormone levels that increases water reabsorption and decreases sodium retention causing hyponatremia. Urea is less likely to cause acute intravascular volume overload. It is more commonly used to treat hyponatremia in cases of the euvolemic form of HF, where no increased blood volume levels are present, instead of the hypervolemic form related to severe cases of HF. The first attempts in 1925 of urea treatment in HF demonstrated increased urine output [42]. However, patients with conditions such as liver toxicity are unable to use this as a therapeutic option in advanced HF.

## Conclusions

Diuretic resistance has been a continuous and long-standing issue in HF patients due to the progressive and interactive effects of decreased cardiac output, decreased GFR, decreased filtration of sodium, and increased tubular reabsorption of sodium. Understanding the pathophysiology and mechanisms contributing to the resistance associated with diuretics can help in its management and implications for HF patients experiencing this condition.

Based on the results of the present investigation, the following conclusions emerge: (1) chronic or excessive use of diuretics is a major cause of DR that is triggered by the braking phenomenon, (2) NSAID use worsens DR by inhibiting prostaglandin synthesis and diminishing renal blood, (3) bioavailability of orally administered diuretics may be reduced because of delayed gut absorption thereby leading to diuretic resistance, (4) alternative methods can be used to mitigate DR by either increasing the diuretic dose or changing the administration route. Once diuretic resistance has been treated successfully, HF treatment may be optimized by implementing patient-tailored care to improve quality of life and enhance the diuretic response.
